# Is autologous platelet activation the key step in ovarian therapy for fertility recovery and menopause reversal?

**DOI:** 10.37796/2211-8039.1380

**Published:** 2022-12-01

**Authors:** Natalie S. Rickers, E. Scott Sills

**Affiliations:** a Plasma Research Section, FertiGen CAG/Regenerative Biology Group, San Clemente, CA, 92673, USA; b Department of Obstetrics & Gynecology, Palomar Medical Center, Escondido, California, USA

**Keywords:** Menopause, Infertility, Platelets, Activation, Rejuvenation

## Abstract

Platelets are a uniquely mammalian physiologic feature. As the only non-marine vertebrates to experience menopause, humans have a substantial post-reproductive lifespan and are believed to have a limited, non-renewable oocyte supply. Ovarian reserve typically declines after about age 35yrs, marking losses which cannot be recovered by available fertility medications. When *in vitro* fertilization fails due to low or absent ovarian response, gonadotropin adjustments are often ineffectual and if additional oocytes are occasionally harvested, egg quality is usually poor. This problem was confronted by Greek researchers who developed a new surgical method to insert autologous platelet-rich plasma (PRP) into ovaries; the first ovarian PRP success to improve reproductive outcomes was published from Athens in 2016. This innovation influenced later research with condensed platelet-derived growth factors, leading to correction of oocyte ploidy error, normal blastocyst development, and additional term livebirths. Yet women’s health was among the last clinical domains to explore PRP, and its role in ‘ovarian rejuvenation’ remains unsettled. One critical aspect in this procedure is platelet activation, a commonly overlooked step in the cytokine release cascade considered essential for successful transition of undifferentiated ovarian stem cells to an oocyte lineage. Poor activation of platelets thus becomes an unforced error, potentially diminishing or even negating post-treatment ovarian follicular response. To answer this query, relevant theory, current disagreements, and new data on platelet activation are presented, along with clinical challenges for regenerative fertility practice.

## 1. Introduction

In 1882, Giulio Bizzozero was examining blood droplets by microscope and noticed specks resembling small *piatto* behaving with odd clumping properties. His was the first paper to describe ‘platelets’ and brought the term to the medical literature [1,2]. Despite being the smallest of all formed elements in circulation, platelets (PLTs) are outnumbered only by erythrocytes and play a key role in cell signaling, wound healing, and tissue growth [3]. Platelet-rich plasma (PRP) is perhaps today the platelet product most familiar to reproductive practice, although it remains an incompletely tested and controversial adjunct before *in vitro* fertilization (IVF). To be sure, its broader acceptance has been slowed by PRP protocols which still require standardization [4] as even subtle change in technique or equipment can yield heterogeneous or indeterminate effects for target tissue. While PLTs contain many growth factors to manage cell differentiation, recruitment and migration as well as directing tissue repair after injury [5], decades of research in hematology, oncology and immunology, find the cytokine portfolio carried by PLTs insufficiently audited. This diverse signaling fund was untapped for IVF until Pantos et al. [6] reported the first PRP success in a human fertility context. Since there are several ways to process and administer PRP [7–9] it is not surprising that diverse responses have accompanied its use in reproductive practice. However, much can be learned from allied clinical and laboratory experience where PLT handling has been better standardized.

## 2. Origins in blood banking

Most published PRP work in fertility practice involve fresh whole blood collection with insertion of the processed sample into ovarian tissue soon after preparation. In contrast, dermatology, cosmetic surgery, and other clinical settings have described freezing PLTs (or PRP) for later use, thus increasing patient convenience by obviating the need for separate blood draws with later treatments. This method of PLT handling and cryostorage has been used by hospitals and blood banks for many years, where resting PLTs (non-activated) are preferred as biological activity starts with PLT activation. This inert status will change only when an urgent clinical need arises, such that PLT activation is not already underway while on the freezer shelf [10]. This principle has relevance for fertility practice as well, where activation should occur just before planned ultrasound-guided ovarian injection [11].

How centrifugation, banking, and storage methods impact frozen PLT function post-thaw has been assessed by comparing PLT extracellular vesicles generated over 2 vs. 7 days [12]. With a longer seven-day storage interval, numerous proteins which attract monocytes and neutrophils were significantly upregulated along with much higher lactate production and deregulation of PLT metabolism [12]. It may be that further investigations discover better protocols for intraovarian PRP where samples are frozen/stored well in advance of injection, but for now there seems to be general agreement that fresh is best.

## 3. Development and anatomy

Platelets are a distinctly mammalian entity. For humans, they are 2–3 μm discoid cytoplasmic fragments with no nucleus, originating from splintered megakaryocytes. PLTs circulate for 7–10 days and it is during this relatively short time that growth factors may be released. Once the PLT lineage is committed, megakaryocyte progenitors undergo endomitosis and polyploidization where numerous DNA replications yield polyploid cells. Supporting this developmental process are integrin receptors which enable communication between the extracellular environment and cell cytoskeleton. Integrins support PLT development with heterodimers of α and β subunits, conferring receptor specificity [13]. Despite being anucleate, PLTs do have residual mRNA and translation enzymes needed for protein synthesis which is left over from their megakaryocyte ancestor [14]. PLTs thus offer a stable proteome for transcription and ribosomal translation analysis [15,16].

## 4. Receptors and release products

**Fig. 1:**
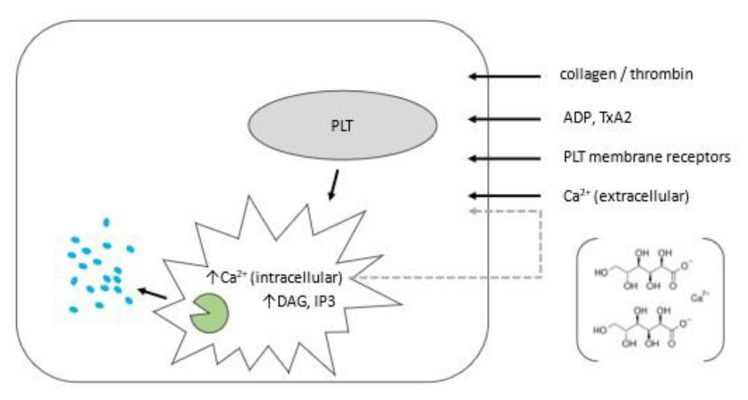
Schema summarizing signaling dynamics for resting (grey oval) and activated platelets (PLT) following 3 response to agonists (i.e., collagen, thrombin), thromboxane A2 (TxA2), adenosine diphosphate (ADP), and/or. 4 extracellular calcium (Ca^2+^). These mediators contribute to increased PLT 1,2-diacylglycerol (DAG) and inositol 5 1,4,5-triphosphate (IP3). Intracellular PLT Ca^2+^ results in calcium efflux (grey arrow), itself augmented by 6 pharmacologic calcium gluconate (bracket) as activator reagent. These mediators coordinate PLT alpha-granule 7 (green) action to discharge ‘cargo proteins’ (blue) including growth factors & cytokines.

Activation of PLTs is the summation of a rapid and complex sequence without which full function cannot be achieved [17,18]. It brings a dynamic reshuffle of the PLT cytoskeleton, an event best studied in response to local tissue injury. Strong cellular–extracellular matrix interactions emerge first, exposed in subendothelial lesions. Of note, recent *in situ* structural study of PLT membrane receptors via high resolution (20–30 Å) cryoelectron tomography detailed an actin filament network able to generate contractile forces required for integrin-mediated functions [19]. The main second messenger here is calcium [20]. Indeed, PLT activation by calcium gluconate induces mass efflux of local growth factors [21] and was the reagent used in the initial ovarian PRP clinical trial in California [11,22]. Calcium gluconate was selected based on contemporary work [23] which found this to be a suitable PLT activator from among several alternatives (see Fig. 1). Interestingly, the first description of a PLT bleeding disorder secondary to altered calcium handling was attributed to a loss-of-function mutation involving a single amino acid shift (D84G) in Sax protein—named for Bulgarian ex- King and Prime Minister Simeon Saxe- Coburg-Gotha (b. 1937) who suffers from the condition [24,25].

PLT activation is also an important defense and repair response, achieved via multiple pathways involving thromboxane A2, adenosine diphosphate, thrombin or other agonists. Any intracellular ‘second message’ received through a stimulated PLT surface antenna (receptor) must be conveyed to a granule-associated protein, which itself is exposed to PLT cytoplasm. Recent research on granule-specific PLT protein processes has highlighted the SNARE family [26] while others have focused on members of the pleckstrin family [27]. Regarding the latter, PLTs without pleckstrin marked impairments are noted in granule exocytosis, actin assembly, and aggregation under certain conditions. While pleckstrin-knockout PLTs can merge granules, they cannot discharge their cargo into the open canalicular system, implicating pleckstrin as a required mediator necessary for granule fusion and exocytosis [28].

Once classified as a waste product of cells, granules are now known to harbor a large repertoire of PLT cargo proteins [29]. Confirmation that PLT α-granules are the key locus of agonist-dependent protein release came from work published in the 1950’s [30]. It is now known that numerous growth factors are released, the most studied of which are probably vascular endothelial growth factor (VEGF), platelet derived growth factor (PDGF), insulin like growth factor-1 (IGF-1), epidermal growth factor (EGF), hepatocyte growth factor (HGF), transforming growth factor beta (TGFβ), and basic fibroblast growth factor (bFGF) [31]. PLT activation involves release from at least two types of subcellular compartments—cell-surface membrane vesicles and exosomes. Experience with PLT processing has shown how both can be sequestered by differential gradient centrifugation. PLT microvesicles of surface membrane class are larger and may be processed using a calcium flux-calpain-dependent protocol [32].

Release of smaller PLT exosomes is by fusion of α-granules with the plasma membrane, where they rest as intraluminal membrane-bound vesicles [32].

The initial phase of PLT-product release brings serotonin and ADP release, followed by a second wave dominated by acid hydrolases and lysosomal proteins [33]. This cascade is based on PLT release kinetics, metabolic characteristics, and subcellular placement of moieties released over time. The mechanism remained only partially understood into the 1970’s, as some aspects were not clearly designated either as an α-granule or dense granule release product [34,35]. Electron microscopy substructure, protein mapping, and later PLT activation experiments offered new data on α-granule anatomy and distribution. For example, a typical resting PLT (non-activated) can include 50–80 ovoid α-granules, each measuring about 200–500 nm diameter [36]. These structures contain adhesive glycoproteins such as P-selectin, fibrinogen, and von Willebrand factor (vWF), coagulation factors, mitogenic factors, angiogenetic factors, fibrinolytic inhibitors, immunoglobulins, granule membrane-specific proteins such as P-selectin and CD63, and chemokines PF4 (also termed CXCL4) and Regulated upon Activation, Normal T-Cell Expressed and Presumably Secreted (RANTES) [37].

In contrast, dense granules (sometimes termed δ-granulomeres) are less numerous within the PLT interior. PLT α-granules have membranes containing p-selectin, an electron-dense luminal matrix and nucleoid, with clustered peripheral tubules of multimeric vWF oligomers [35]. In any case, PLT α-granules likely exhibit some variance among granules. For these to discharge contents at activation, the packet can merge directly with the exterior plasma membrane or with the PLT open canalicular system [38]. This latter structure manifests as complex plasma membrane invaginations to substantially enlarge PLT surface area; de-condensation can be facilitated by contact with the plasma membrane even if the union is transient [39,40].

Data supporting fusion mechanisms comes from electron microscopy work, identifying PLT α-granules fused to the plasma membrane either by a short neck or longer ‘pipe’ [41]. During activation, PLT proteins from αgranules appear to be jettisoned via membrane fusion but in contrast to synaptic vesicles, the PLT α-granule itself is not lost in the process. More exactly, an empty PLT α-granule ‘vestige’ lingers after membrane fusion akin to an opening which connects the granular remnant to the extracellular space [42], an event which may be reversible. Of note, amperage status by membrane electrophysiology shows a capacitance ‘flickering’ as release is underway, possibly indicating that PLT pores and their ghosts are unstable [42].

## 5. Activation: specific or general?

A range of surface receptors suggests that PLTs have flexibility to mount a bespoke response to different agonist inputs, sorting membrane inputs even down to the individual granule level. From a mechanistic viewpoint, there are two different pathways by which PLTs activate—endogenous and exogenous. The first follows vessel injury and contact of PLTs with exposed collagen (physiologic activation). Exogenous activation is via pharmacologic reagents, and those most frequently used are 10% calcium chloride, 10% calcium gluconate, and thrombin. For 10% CaCl2, a dosing of 1:9 (reagent:PRP) is typical and this ratio may also be used with calcium gluconate [43].

Earlier work reported that protease-activated receptor-1 (PAR-1) and PAR-4 agonists counter-regulate endostatin vs. VEGF release from human PLTs [44], although evidence to confirm such differential release from αgranule subtypes is pending. Others [45] have questioned this, observing a kinetic rather than context-driven difference in PLT granule release after exposure to various agonists, a finding which challenges the α-granule subtype theory. One interesting problem confronting this topic is that human PLTs express some receptors on surface membranes (PAR-1 and PAR-4), while most other vertebrates below primates display different exterior receptors [46]. Velez et al. [47] reported almost all differences in PLT release following contact with thrombin vs. collagen resulted from proteolytic activity of thrombin agonist on the releasate. From this, PLT α-granules appeared to respond in concert to agonist stimulation, such that any stimulatory signal was perceived uniformly by the α-granule ensemble [48].

Integrin mediates conformational change [49] needed for signaling and PLT adhesion, even though this has not yet been directly observed in physiologic conditions [19]. PLT deformation utilizes an actomyosin contractile system [50,51] which time-lapse imaging has shown begins with contraction and then a flare of extended pseudopodia, akin to a molecular clutch [52]. In 2009, Kinoshita et al. [53] reported on a previously unknown kinase, vertebrate lonesome kinase (Vlk), which is highly conserved in vertebrates. This kinase domain cannot be classified under any previously defined groups or families, and is now regarded as critical to regulate protein export from the Golgi apparatus and PLT competency in general [53,54]. PLT-derived cytokines stimulate stem cell differentiation, proliferation, and migration to improve angiogenesis and can also modulate inflammation [21,55]. While these cytokines can potentiate recovery by ‘natural healing’, matching results with specific components of PLT releasate is the goal of ongoing study.

Modulating particular members within the cytokine family could influence the prevailing treatment effect [56]. In other words, increasing PLT cytokine output *en gross* is desirable only up to a specified threshold, above which point an accumulation of pro-inflammatory mediators dominates other biological effects. Even at lower concentrations, PRP still shows some chemotactic properties for stem cells [57]. It is notable that when PRP was studied after experimental dilution down to 10% or 20% concentrations [58], the lowest density preparation exhibited better growth and secretion of hepatocyte growth factor, monocyte chemoattractant protein-1, epithelial neutrophil-activating peptide-78, and VEGF. Moreover, the 20% PRP culture showed significantly higher levels of pro-inflammatory granulocyte-macrophage colony-stimulating factor [58]. He et al. [59] hypothesized that an undesirable cytokine profile is related to leukocyte load, and PRP handling technique can influence this leukocyte accumulation. Indeed, PLT derivatives with many leukocytes are more often associated with growth factor suppression, decreased cell viability, increased catabolic cytokine concentrations, and inflammation [60].

When two PRP preparations differing only in leukocyte content were assessed [61], samples with more leukocytes led to catabolic and inflammatory changes and overexpression of catabolic marker genes, matrix metalloproteinase-1 (MMP-1), MMP-13, interleukin-1beta (IL-1β), IL-6 and tumor necrosis factor-alpha (TNF-α). In contrast, PRP samples with reduced leukocyte counts chiefly induced anabolic changes [61]. While the optimal balance between PLTs and leukocytes for reproductive use has not been established, a relatively enriched PLT concentration appears favorable. We therefore agree with previous work [62] which found that minimizing leukocytes in PRP is preferred over simply boosting absolute PLT numbers.

It may be that individual PRP cytokines evoke narrow action upon specific cell populations with comparable, perhaps greater, efficiency than unprocessed PRP. Depending on how PLTs are cultured or supported before tissue insertion, cell death is far more likely when PRP concentration is excessive (i.e., ≥50%), underscoring a potential role for PLT factor adjustment according to indication [59]. A negative tissue response post-PRP might result from excessive osmotic pressure or an uptick of proinflammatory mediators, both increasing ROS and favoring apoptosis [5]. Suboptimal PRP effects have also been attributed to elevated Fas ligand (FasL), IL-1β, IL-6 and TNFα levels [63]. Another inflammatory cytokine is interferon-γ, which accelerates cellular apoptosis when overproduced [64]. As a corrective technique, PRP immunoprecipitation might cancel such levels by silencing unhelpful pro-inflammatory mediators, thus providing a custom PRP product [59].

Since PLT cargo proteins are lodged inside granules and thus maintain latency until activation, none of these signaling factors attain any relevance to the ovary until the critical step of activation. Research regarding how this process is orchestrated suggests that ultrarapid ‘crescendo’ PLT activation is non-physiologic [59]. This raises an unusual technical question given the impressive rate at which PLT activation normally proceeds. Yang et al. [65] reported on PLT activation by calcium gluconate where slow, gradual release of cargo proteins was observed. Rui et al. [66] found that activation by thrombin +calcium gluconate produced the highest growth factor concentration at PLT activation. Cytokines were also noted to be released differentially from PLT granules, according to specific activation signaling features [66]. Whether or not these identifiers can be actuated for preferential discharge of specified PLT factors upon ‘surgical activation’ remains to be proven.

## 6. Other variables

Screening for intraovarian PRP must query patients on medication and botanical use, as PLT activation will be reduced or blocked by several compounds. The most familiar agent with potent anti-PLT action is aspirin, although pentoxifylline should probably also be avoided for 10–14 days before planned PRP use [67]. Turmeric is a cooking spice and health supplement with important effects on PLTs. Specifically, tetrahydrocurcumin is the major bioactive metabolite of curcumin and significantly attenuates agonist-induced PLT granule secretion [68]. Safflower extract likewise inhibits ADP-mediated PLT function, impairing activation of downstream ADP receptors [69].

Ambient physiologic conditions can also influence what PLTs release after activation. Altered membrane lipids and proteins exist in hypertension, impacting PLT function and leading to formation of vascular obstruction. Armenta-Medina et al. [70] identified numerous PLT proteins expressed differently, depending on blood pressure. Hypertension thus can influence PLT cytoskeletal organization and release of granule cargo [70] which can show a wide range in total protein, fibrinogen, albumin, IgG, as well as antioxidative capacity [71]. Recent data from Geneva outlined a method where after centrifugation, blood components were separated to isolate PRP and was considered better than standard media to support and expand human cell culture [72]. Previously, two distinct PLT protocols which otherwise used identical equipment were compared and found to produce significantly different results, especially regarding matrix leukocytes and growth factors [73].

Higher levels of fibrinogen, albumin, IGF-1, keratinocyte growth factor (KGF), and HGF often occur in samples which include plasma. Conversely, chemokine RANTES and PDGF-bb were increased with PLT proteins. Specimens containing both plasma and plasma proteins have the most pronounced proliferation effects for mesenchymal stem cells & fibroblasts [74].

## 7. Conclusions

Gains in longevity now make menopause relevant for about 1/3rd of the female lifespan, and by 2025 about one billion women will experience this journey worldwide. While differences among published ovarian PRP methods may meet some needs of advancing age, when successful they all have the common attribute of clearly delineated PLT activation. For ‘ovarian rejuvenation’, local cross-talk between PLT cytokines and downstream ovarian stem cells is obligatory. Indeed, this was foreshadowed by work from Pantos et al. [6] which presaged a seismic shift in women’s health and fertility practice. Fundamentally this PRP-based treatment aspires to realign the ovarian microclimate, even if only temporarily, to conditions lost with the descent into menopause. True, a remedy is already reachable to some extent using hormone replacement therapy or IVF. Yet with activated PLTs, a nonsynthetic, personalized dimension to care can alleviate concerns with ‘artificial’ pharmaceuticals engineered at industrial scale. Questions certainly remain for intraovarian PRP, and researchers are exploring these topics. For example, the potential for PRP to induce tumorigenic transformation requires an answer. This appears to be a theoretical issue only, as such danger has fortunately not been observed in any tissue context where PRP has been used. The likely explanation for this is that PLT cytokines bind to receptors on cell membranes—not in the nucleus—so their mechanism of action is unlike trophic hormones [55]. Reassurance also comes from others using PRP outside reproductive medicine, where accelerated growth of healthy cells is attained with no malignant transformation [75]. In vitro assays have likewise shown PLTs block the tendency of cancer cells to establish ‘vasculogenic mimicry’ or to acquire independent vessel connections [76]. These features help explain why intraovarian PRP methods, irrespective of which protocol is used, thus far have proven safe with no adverse events [77].

## References

[1] Brewer DB Max Schultze (1865), G. Bizzozero (1882) and the discovery of the platelet Br J Haematol 2006 133 3 251 8 10.1111/j.1365-2141.2006.06036.x 16643426

[2] Ribatti D Crivellato E Giulio Bizzozero and the discovery of platelets Leuk Res 2007 31 10 1339 41 10.1016/j.leukres.2007.02.008 17383722

[3] Gremmel T Frelinger AL 3rd Michelson AD Platelet physiology Semin Thromb Hemost 2016 42 3 191 204 10.1055/s-0035-1564835 26926581

[4] Schär MO Diaz-Romero J Kohl S Zumstein MA Nesic D Platelet-rich concentrates differentially release growth factors and induce cell migration in vitro Clin Orthop Relat Res 2015 473 5 1635 43 10.1007/s11999-015-4192-2 25690170 PMC4385378

[5] Wood SH Sills ES Intraovarian vascular enhancement via stromal injection of platelet-derived growth factors: exploring subsequent oocyte chromosomal status and in vitro fertilization outcomes Clin Exp Reprod Med 2020 47 2 94 100 10.5653/cerm.2019.03405 32466629 PMC7315860

[6] Pantos K Nitsos N Kokkali G Vaxevanoglou T Markomichali C Pantou A Ovarian rejuvenation and folliculogenesis reactivation in peri-menopausal women after autologous platelet-rich plasma treatment [*abstract*] Hum Reprod 2016 401 https://sa1s3.patientpop.com/assets/docs/111052.pdf

[7] Pantou A Giannelou P Grigoriadis S Maziotis E Tzonis P Koutsouni A Evaluating different strategies for poor ovarian response management: a retrospective cohort study and literature review Ann N Y Acad Sci 2021 1500 1 93 111 10.1111/nyas.14614 34046896

[8] Petryk N Petryk M Ovarian rejuvenation through platelet-rich autologous plasma (PRP) - a chance to have a baby without donor eggs, improving the life quality of women suffering from early menopause without synthetic hormonal treatment Reprod Sci 2020 27 11 1975 82 10.1007/s43032-020-00266-8 32700285

[9] Ferrari AR Cortrezzi S Borges E Jr Braga D Souza MDCB Antunes RA Evaluation of effects of platelet-rich plasma on follicular and endometrial growth: a literature review JBRA Assist Reprod 2021 25 4 601 7 10.5935/1518-0557.20210036 34415119 PMC8489815

[10] Dubiel B Resting vs. activated platelets: why knowing the difference makes financial sense Hematol Advisor [newspaper] November 14 2019 https://www.hematologyadvisor.com/home/topics/bleedingdisorders/improving-financial-and-health-outcomes-by-classifying-resting-and-activated-platelets/

[11] Sills ES Rickers NS Li X Palermo GD First data on in vitro fertilization and blastocyst formation after intraovarian injection of calcium gluconate-activated autologous platelet rich plasma Gynecol Endocrinol 2018 34 9 756 60 10.1080/09513590.2018.1445219 29486615

[12] Hermida-Nogueira L Barrachina MN Izquierdo I García-Vence M Lacerenza S Bravo S Proteomic analysis of extracellular vesicles derived from platelet concentrates treated with Mirasol® identifies biomarkers of platelet storage lesion J Proteonomics 2020 210 103529 10.1016/j.jprot.2019.103529 31605789

[13] Yang X Chitalia SV Matsuura S Ravid K Integrins and their role in megakaryocyte development and function Exp Hematol 2021 S0301-472X 21 625 31 10.1016/j.exphem.2021.11.007 PMC8795491 34910941

[14] Nassa G Giurato G Cimmino G Rizzo F Ravo M Salvati A Splicing of platelet resident premRNAs upon activation by physiological stimuli results in functionally relevant proteome modifications Sci Rep 2018 8 1 498 10.1038/s41598-017-18985-5 29323256 PMC5765118

[15] Looße C Swieringa F Heemskerk JWM Sickmann A Lorenz C Platelet proteomics: from discovery to diagnosis Expert Rev Proteomics 2018 15 6 467 76 10.1080/14789450.2018.1480111 29787335

[16] Huang J Zhang P Solari FA Sickmann A Garcia A Jurk K Molecular proteomics and signaling of human platelets in health and disease Int J Mol Sci 2021 22 18 9860 10.3390/ijms22189860 34576024 PMC8468031

[17] Heijnen HF Debili N Vainchencker W Breton-Gorius J Geuze HJ Sixma JJ Multivesicular bodies are an intermediate stage in the formation of platelet alpha-granules Blood 1998 91 7 2313 25 https://pubmed.ncbi.nlm.nih.gov/9516129/ 9516129

[18] Silva RF Alvarez ME Ríos DL López C Carmona JU Rezende CM Evaluation of the effect of calcium gluconate and bovine thrombin on the temporal release of transforming growth factor beta 1 and plateletderived growth factor isoform BB from feline platelet concentrates BMC Vet Res 2012 8 212 10.1186/1746-6148-8-212 23131192 PMC3534502

[19] Sorrentino S Conesa JJ Cuervo A Melero R Martins B Fernandez-Gimenez E Structural analysis of receptors and actin polarity in platelet protrusions Proc Natl Acad Sci U S A 2021 118 37 e2105004118 10.1073/pnas.2105004118 34504018 PMC8449362

[20] Cavallo C Roffi A Grigolo B Mariani E Pratelli L Merli G Platelet-rich plasma: the choice of activation method affects the release of bioactive molecules BioMed Res Int 2016 2016 6591717 10.1155/2016/6591717 PMC5031826 27672658

[21] Margono A Bagio DA Julianto I Suprastiwi E The effect of calcium gluconate on platelet rich plasma activation for VEGF-A expression of human dental pulp stem cells Eur J Dermatol 2021 Dec 22 10.1055/s-0041-1735930 PMC9339933 34937106

[22] Sills ES Rickers NS Petersen JL Li X Wood SH Regenerative effect of intraovarian injection of autologous platelet rich plasma: serum anti-Mullerian hormone levels measured among poor-prognosis in vitro fertilization patients Int J Regen Med 2020 3 1 5 10.31487/j.RGM.2020.01.02

[23] Giraldo CE Álvarez ME Carmona JU Influence of calcium salts and bovine thrombin on growth factor release from equine platelet-rich gel supernatants Vet Comp Orthop Traumatol 2017 30 1 1 7 10.3415/VCOT-16-02-0026 27849108

[24] Grosse J Braun A Varga-Szabo D Beyersdorf N Schneider B Zeitlmann L An EF hand mutation in Stim1 causes premature platelet activation and bleeding in mice J Clin Invest 2007 117 11 3540 50 10.1172/JCI32312 17965774 PMC2040319

[25] Varga-Szabo D Braun A Nieswandt B Calcium signaling in platelets J Thromb Haemostasis 2009 7 7 1057 66 10.1111/j.1538-7836.2009.03455.x 19422456

[26] Peters CG Michelson AD Flaumenhaft R Granule exocytosis is required for platelet spreading: differential sorting of α-granules expressing VAMP-7 Blood 2012 120 1 19 10.1182/blood-2011-10-389247 PMC3390958 22589474

[27] Wang G Zhou Q Xu Y Zhao B Emerging roles of Pleckstrin-2 beyond cell spreading Front Cell Dev Biol 2021 9 768238 10.3389/fcell.2021.768238 34869363 PMC8637889

[28] Lian L Wang Y Flick M Choi J Scott EW Degen J Loss of Pleckstrin defines a novel pathway for PKC-mediated exocytosis Blood 2009 113 15 3577 84 19190246 10.1182/blood-2008-09-178913 PMC2668855

[29] Chen Y Zhao Y Yin Y Jia X Mao L Mechanism of cargo sorting into small extracellular vesicles Bioengineered 2021 12 1 8186 201 10.1080/21655979.2021.1977767 34661500 PMC8806638

[30] Zucker MB Borrelli J Quantity, assay, and release of serotonin in human platelets J Appl Physiol 1955 7 4 425 31 10.1152/jappl.1955.7.4.425 13233134

[31] Cecerska-Heryć E Goszka M Serwin N Roszak M Grygorcewicz B Heryć R Applications of the regenerative capacity of platelets in modern medicine Cytokine Growth Factor Rev 2021 S1359– 6101 21 90 3 10.1016/j.cytogfr.2021.11.003 34924312

[32] Janowska-Wieczorek A Wysoczynski M Kijowski J Marquez-Curtis L Machalinski B Ratajczak J Microvesicles derived from activated platelets induce metastasis and angiogenesis in lung cancer Int J Cancer 2005 113 752 60 10.1002/ijc.20657 15499615

[33] Lages B Scrutton MC Holmsen H Studies on gel-filtered human platelets: isolation and characterization in a medium containing no added Ca2+, Mg2+, or K+ J Lab Clin Med 1975 85 5 811 25 https://pubmed.ncbi.nlm.nih.gov/164512/ 164512

[34] Walsh PN Gagnatelli G Platelet antiheparin activity: storage site and release mechanism Blood 1974 44 2 157 68 https://pubmed.ncbi.nlm.nih.gov/4604917/ 4604917

[35] Yadav S Storrie B The cellular basis of platelet secretion: emerging structure/function relationships Platelets 2017 28 2 108 18 10.1080/09537104.2016.1257786 28010140 PMC5627609

[36] King SM Reed GL Development of platelet secretory granules Semin Cell Dev Biol 2002 13 4 293 302 10.1016/s1084952102000599 12243729

[37] Yun SH Sim EH Goh RY Park JI Han JY Platelet activation: the mechanisms and potential biomarkers BioMed Res Int 2016 2016 9060143 10.1155/2016/9060143 PMC4925965 27403440

[38] Selvadurai MV Hamilton JR Structure and function of the open canalicular system - the platelet’s specialized internal membrane network Platelets 2018 29 4 319 25 10.1080/09537104.2018.1431388 29442528

[39] Howell K White JG Hobert O Spatiotemporal control of a novel synaptic organizer molecule Nature 2015 523 7558 83 7 10.1038/nature14545 26083757 PMC9134992

[40] Won E Morodomi Y Kanaji S Shapiro R Vo MN Orje JN Extracellular tyrosyl-tRNA synthetase cleaved by plasma proteinases and stored in platelet α-granules: potential role in monocyte activation Res Pract Thromb Haemost 2020 4 7 1167 77 10.1002/rth2.12429 33134783 PMC7590329

[41] Mammadova-Bach E Braun A Platelet life without TMEM163: No dense granules Blood 2021 137 13 1708 9 10.1182/blood.2021010691 33792677

[42] Koseoglu S Dilks JR Peters CG Fitch-Tewfik JL Fadel NA Jasuja R Dynamin-related protein-1 controls fusion pore dynamics during platelet granule exocytosis Arterioscler Thromb Vasc Biol 2013 33 3 481 8 10.1161/ATVBAHA.112.255737 23288151 PMC3573216

[43] Sills ES Rickers NS Wood SH Intraovarian insertion of autologous platelet growth factors as cell-free concentrate: fertility recovery and first unassisted conception with term delivery at age over 40 Int J Reprod Biomed 2020 18 12 1081 6 10.18502/ijrm.v18i12.8030 33426419 PMC7778756

[44] Ma L Perini R McKnight W Dicay M Klein A Hollenberg MD Proteinase-activated receptors 1 and 4 counter-regulate endostatin and VEGF release from human platelets Proc Natl Acad Sci U S A 2005 102 1 216 20 10.1073/pnas.0406682102 15615851 PMC544057

[45] Jonnalagadda D Izu LT Whiteheart SW Platelet secretion is kinetically heterogeneous in an agonistresponsive manner Blood 2012 120 26 5209 16 10.1182/blood-2012-07-445080 23086755 PMC3537312

[46] Ossovskaya VS Bunnett NW Protease-activated receptors: contribution to physiology and disease Physiol Rev 2004 84 2 579 621 10.1152/physrev.00028.2003 15044683

[47] Vélez P Izquierdo I Rosa I García Á A 2D–DIGE-based proteomic analysis reveals differences in the platelet releasate composition when comparing thrombin and collagen stimulations Sci Rep 2015 5 8198 10.1038/srep08198 25645904 PMC4316189

[48] Heinzmann ACA Coenen DM Vajen T Cosemans JMEM Koenen RR Combined antiplatelet therapy reduces the proinflammatory properties of activated platelets TH Open 2021 5 4 e533 42 10.1055/a-1682-3415 34901735 PMC8651446

[49] Xu XP Kim E Swift M Smith JW Volkmann N Hanein D Three-Dimensional structures of full-length, membrane-embedded human α(IIb)β(3) integrin complexes Biophys J 2016 110 4 798 809 10.1016/j.bpj.2016.01.016 26910421 PMC4776043

[50] Paknikar AK Eltzner B Köster S Direct characterization of cytoskeletal reorganization during blood platelet spreading Prog Biophys Mol Biol 2019 144 166 76 10.1016/j.pbiomolbio.2018.05.001 29843920

[51] Cheng B Yan R Zhang SQ Yang MN Dai KS The role of zyxin in regulating platelet cytoskeleton distribution Zhongguo Shi Yan Xue Ye Xue Za Zhi 2021 29 3 876 80 10.19746/j.cnki.issn.1009-2137.2021.03.035 34105487

[52] Owen LM Adhikari AS Patel M Grimmer P Leijnse N Kim MC A cytoskeletal clutch mediates cellular force transmission in a soft, three-dimensional extracellular matrix Mol Biol Cell 2017 28 14 1959 74 10.1091/mbc.E17-02-0102 28592635 PMC5541846

[53] Kinoshita M Era T Jakt LM Nishikawa S The novel protein kinase Vlk is essential for stromal function of mesenchymal cells Development 2009 136 12 2069 79 10.1242/dev.026435 19465597

[54] Revollo LD Merrill-Skoloff G De Ceunynck K Dilks JR Guo S Bordoli MR The secreted tyrosine kinase VLK is essential for normal platelet activation and thrombus formation Blood 2021 10.1182/blood.2020010342.blood PMC8718620 34329392

[55] Sills ES Wood SH Autologous activated platelet-rich plasma injection into adult human ovary tissue: molecular mechanism, analysis, and discussion of reproductive response Biosci Rep 2019 39 6 BSR20190805 10.1042/BSR20190805 PMC6549090 31092698

[56] Yin W Qi X Zhang Y Sheng J Xu Z Tao S Advantages of pure platelet-rich plasma compared with leukocyte- and platelet-rich plasma in promoting repair of bone defects J Transl Med 2016 14 73 10.1186/s12967-016-0825-9 26980293 PMC4792107

[57] LaPrade RF Goodrich LR Phillips J Dornan GJ Turnbull TL Hawes ML Use of platelet-rich plasma immediately after an injury did not improve ligament healing, and increasing platelet concentrations was detrimental in an *in vivo* animal model Am J Sports Med 2018 46 3 702 12 10.1177/0363546517741135 29211969

[58] Xian LJ Chowdhury SR Bin Saim A Idrus RB Concentration-dependent effect of platelet-rich plasma on keratinocyte and fibroblast wound healing Cytotherapy 2015 17 3 293 300 10.1016/j.jcyt.2014.10.005 25456581

[59] He L Gong Q Ahn JA Chotkowski G Bai H Pei J Mechanistic insight of platelet-rich plasma (PRP) in mesenchymal and vascular progenitors DOI: 10.2139/ssrn.3244937

[60] Zhang L Chen S Chang P Bao N Yang C Ti Y Harmful effects of leukocyte-rich platelet-rich plasma on rabbit tendon stem cells in vitro Am J Sports Med 2016 44 8 1941 51 10.1177/0363546516644718 27184544

[61] Zhou Y Zhang J Wu H Hogan MV Wang JH The differential effects of leukocyte-containing and pure platelet-rich plasma (PRP) on tendon stem/progenitor cells - implications of PRP application for the clinical treatment of tendon injuries Stem Cell Res Ther 2015 6 1 173 10.1186/s132872-4 26373929 PMC4572462

[62] Boswell SG Schnabel LV Mohammed HO Sundman EA Minas T Fortier LA Increasing platelet concentrations in leukocyte-reduced platelet-rich plasma decrease collagen gene synthesis in tendons Am J Sports Med 2014 42 1 42 9 10.1177/0363546513507566 24136860

[63] Hudgens JL Sugg KB Grekin JA Gumucio JP Bedi A Mendias CL Platelet-rich plasma activates proinflammatory signaling pathways and induces oxidative stress in tendon fibroblasts Am J Sports Med 2016 44 8 1931 40 10.1177/0363546516637176 27400714 PMC4970921

[64] Wu H Wang L Zhang D Qian J Yan L Tang Q PRDM5 promotes the apoptosis of epithelial cells induced by IFN-γ during Crohn’s disease Pathol Res Pract 2017 213 6 666 73 10.1016/j.prp.2016.12.004 28476379

[65] Yang Y Zhang W Cheng B Experimental research on the effects of different activators on the formation of platelet-rich gel and the release of bioactive substances in human platelet-rich plasma Zhonghua Shaoshang Zazhi 2017 33 1 12 7 10.3760/cma.j.issn.1009-2587.2017.01.004 28103989

[66] Rui S Yuan Y Du C Song P Chen Y Wang H Comparison and investigation of exosomes derived from platelet-rich plasma activated by different agonists Cell Transplant 2021 30 9636897211017833 10.1177/09636897211017833 34006140 PMC8138303

[67] Sills ES Why might ovarian rejuvenation fail? Decision analysis of variables impacting reproductive response after autologous platelet-rich plasma Minerva Obstet Gynecol 2022 Feb 2 10.23736/S2724-606X.22.04996-X 35107239

[68] Li W Ma Y Zhang C Chen B Zhang X Yu X Tetrahydrocurcumin downregulates MAPKs/cPLA2 signaling and attenuates platelet thromboxane A2 generation, granule secretion, and thrombus growth Thromb Haemostasis 2021 Aug 24 10.1055/s-0041-1735192 34428833

[69] Lu PH Kuo CY Chan CC Wang LK Chen ML Tzeng IS Safflower extract inhibits ADP-induced human platelet aggregation Plants (Basel) 2021 10 6 1192 10.3390/plants10061192 34208125 PMC8230796

[70] Armenta-Medina Y Martínez-Vieyra I Medina-Contreras O Benitez-Cardoza CG Jiménez-Pineda A Reyes-López CA Differentially expressed proteins in platelets derived from patients with hypertension J Hum Hypertens 2021 1 11 10.1038/s41371-021-00555-y PMC8254060 34218268

[71] Delila L Wu YW Nebie O Widyaningrum R Chou ML Devos D Extensive characterization of the composition and functional activities of five preparations of human platelet lysates for dedicated clinical uses Platelets 2021 32 2 259 72 10.1080/09537104.2020.1849603 33245683

[72] Berndt S Turzi A Modarressi A Production of autologous platelet-rich plasma for boosting in vitro human fibroblast expansion JoVE 2021 168 10.3791/60816 33720140

[73] Dohan Ehrenfest DM Pinto NR Pereda A Jiménez P Corso MD Kang BS The impact of the centrifuge characteristics and centrifugation protocols on the cells, growth factors, and fibrin architecture of a leukocyte- and platelet-rich fibrin (L-PRF) clot and membrane Platelets 2018 29 2 171 84 10.1080/09537104.2017.1293812 28437133

[74] Sovkova V Vocetkova K Hedvičáková V Hefka Blahnová V Buzgo M Amler E Cellular response to individual components of the platelet concentrate Int J Mol Sci 2021 22 9 4539 10.3390/ijms22094539 33926125 PMC8123700

[75] García-Martínez O Reyes-Botella C Díaz-Rodríguez L De Luna-Bertos E Ramos-Torrecillas J Vallecillo-Capilla MF Effect of platelet-rich plasma on growth and antigenic profile of human osteoblasts and its clinical impact J Oral Maxillofac Surg 2012 70 7 1558 64 10.1016/j.joms.2011.06.199 21864971

[76] Martini C Thompson EJ Hyslop SR Cockshell MP Dale BJ Ebert LM Platelets disrupt vasculogenic mimicry by cancer cells Sci Rep 2020 10 1 5869 10.1038/s41598-020-62648-x 32246008 PMC7125143

[77] Sills ES The scientific and cultural journey to ovarian rejuvenation: background, barriers, and beyond the biological clock Medicines (Basel) 2021 8 6 29 10.3390/medicines8060029 34201170 PMC8228162

